# Allergen immunotherapy for the control of moderate to severe allergic asthma: an evidence-based conjoint analysis to define candidate patient profiles in Spain and Portugal

**DOI:** 10.3389/falgy.2025.1676399

**Published:** 2025-10-30

**Authors:** J. Delgado, R. Cárdenas, S. Gelis, J. Domínguez-Ortega

**Affiliations:** 1 Allergy Department, Hospital Virgen Macarena, Sevilla, Spain; 2 Allergy Department, Hospital Universitario Virgen del Rocío, Sevilla, Spain; 3 Allergy Department, Hospital Clínic, Institut d'Investigacions Biomèdiques August Pi i Sunyer (IDIBAPS), Barcelona, Spain; 4 Allergy Department, Hospital Universitario La Paz, Institute for Health Research IDiPAZ, Madrid, Spain

**Keywords:** moderate to severe allergic asthma, allergen immunotherapy, AIT, conjoint analysis, patient profiling, etiological treatment

## Abstract

**Introduction:**

Allergen immunotherapy (AIT) is an effective and safe treatment; however, it is not recommended in consensus guidelines for severe allergic asthma patients. As AIT has been shown to be capable of modifying the course of the disease, it should be considered a concomitant treatment for specific asthma patients. This study aimed to define the profile of patients with severe allergic asthma who are most likely to benefit from AIT.

**Methods:**

A conjoint analysis approach was adopted to comprehensively assess the importance of clinical attributes in therapeutic decision-making. A scientific committee selected the main attributes to be considered: lung function, clinical control of allergic asthma, current main treatment and etiological confirmation of moderate to severe allergic asthma. Using the fractional factorial analysis technique, 8 eligible patient profiles for AIT were defined. Participant allergists, by means of a questionnaire, classified the profiles in order of preference, mimicking the comprehensive assessment performed in clinical practice.

**Results:**

91 allergists from Spain and Portugal with experience in asthma and AIT participated in the study. Allergists gave greater importance to the clinical control of allergic asthma (relative importance of 51.6%), followed by preserved lung function (relative importance of 25.0%), thus confirming that the most important criterion was good control of the underlying asthmatic condition.

**Conclusions:**

The expert allergists endorse the use of AIT in the management of moderate to severe allergic asthma in patients with appropriate clinical characteristics. Additional studies to further investigate the safety and effectiveness of this new therapeutic approach would be of interest.

## Introduction

Asthma affects more than 350 million people of all ages worldwide, and its prevalence is increasing. Furthermore, asthma is the second leading cause of mortality among chronic respiratory diseases, accounting for an estimated 436,193 deaths worldwide in 2021 (95% UI, 357,795–555,604), which makes it a serious global health concern (1, 2). It is a chronic and heterogeneous lung disease characterized by chronic inflammation and narrowing of the airways. The main clinical manifestations include respiratory symptoms, such as wheezing, coughing, chest tightness, and shortness of breath (2).

There are several clinical asthma phenotypes, with allergic asthma being the most common (2, 3). Allergic asthma is characterized by allergen-specific immunoglobulin E (sIgE) sensitization with symptoms triggered by allergen exposure (4). Since IgE is the key mediator in allergic asthma, it has become an important therapeutic target.

In this context, allergen immunotherapy (AIT), which involves the repeated administration of allergen extracts, represents a valuable therapeutic tool, as it addresses the underlying cause of the disease (5, 6). AIT is the only etiological treatment capable of modifying the natural course of the disease, as it induces a shift from Th2 to Th1 response eventually leading to IgE suppression (5, 7). AIT improves asthma symptoms with short- and long-term benefits, reduction in medication use and better disease control, thus preventing disease worsening. Additionally, when used in combination with biologic therapies, AIT improves both efficacy and safety and facilitates asthma control (6, 7).

The key component in asthma management consists of pharmacological treatment, in stepwise approach, mainly medium or high doses of inhaled corticosteroids (ICS) alone, commonly associated with long-acting *β*_2_ agonist (LABA) in increasing doses according to disease severity, and frequently, long-acting muscarinic agents (LAMA) and/or antileukotrienes. In some cases, biologic treatment is used to prevent the use of oral corticosteroids (OCS) and exacerbations.

The current Spanish Guideline on the Management of Asthma (GEMA 5.4) classifies patients on steps 5 and 6 as severe asthma, while in the Global Initiative for Asthma (GINA 2024) steps 4–5 are not so specific (2, 8). According to GEMA 5.4, AIT is recommended for patients with well-controlled allergic asthma on low or medium step treatment (steps 2–4 GEMA 5.4 and steps 1–3 GINA) and clinically relevant IgE-mediated sensitization to common aeroallergens (2, 8). Allergen immunotherapy has been acknowledged by international expert bodies as add-on treatment for mild/moderate asthma (9), its safety and cost-effectiveness has been demonstrated in these more severe cases (10). However, the profile of patients with moderate-to-severe allergic asthma who are candidates for AIT is not well defined. Therefore, the aim of the present study was to evaluate the attributes most highly valued by allergists when considering AIT in patients with moderate to severe asthma.

This work is exploratory and descriptive in nature, designed to generate preliminary real-world evidence to inform future research and guideline discussions.

## Methods

In the present study, a conjoint value analysis (CVA) methodology was adopted to rigorously elicit preferences (11) regarding the prescription of Allergen Immunotherapy (AIT). This methodology was adopted to estimate the relative importance of clinical characteristics, defined by specific attributes and levels when considering patients with moderate to severe allergic asthma. The assessment involved presenting complete patient profiles, which participants ranked according to preference. This study is exploratory and descriptive in nature, and the findings should be interpreted as preliminary evidence.

### Committee

The study was led, designed and performed by the Steering Committee, which comprised four allergy specialists with recognized authority and extensive expertise in allergology, particularly in managing moderate to severe allergic asthma. The Committee was also responsible for conceptualizing and preparing the questionnaire that was subsequently completed by the participating allergists.

### Participant recruitment

The present study included allergists who routinely treat asthma patients, working in health care centers from Spain and Portugal. Non-specialists in the allergy field or specialists working in other countries were excluded. The participants were recruited between November 18, 2024, and January 13, 2025, and all of them provided their consent prior to participation. A total of 91 allergists participated in the conjoint analysis questionnaire.

### Questionnaire design

#### Attributes

Based on a literature review and their vast experience, the SC selected the main attributes to be considered in AIT decision-making for moderate to severe allergic asthma:

1. Lung function.
2. Clinical control of allergic asthma.
3. Current main treatment of choice for moderate to severe allergic asthma.
4. Etiological confirmation of moderate to severe allergic asthma.

#### Attribute levels

The number of levels for each attribute was limited to two or three, according to the International Society for Pharmacoeconomics and Outcomes Research (ISPOR) Good Research Practices for Conjoint Analysis Task Force recommendations (12). The specific levels defined were (Table 1):

- Lung function: Forced Expiratory Volume in 1 second (FEV₁) ≥ 70% or FEV₁ < 70%.
- Method of etiological confirmation: Skin prick test (SPT)/specific IgE or component-resolved diagnosis.
- Clinical control: Totally controlled, partly controlled, or uncontrolled disease.
- Treatment: High-dose ICS + LABA or high-dose ICS + LABA with biologic therapies as an add-on therapy.

**Table 1:** Patient profiles: attributes and levels considered.

Profiles	Profile 1	Profile 2	Profile 3	Profile 4	Profile 5	Profile 6	Profile 7	Profile 8
Attributes								
Method for etiological confirmation of moderate to severe allergic asthma	SPT/specific IgE	Component resolved diagnosis	SPT/specific IgE	SPT/specific IgE	Component resolved diagnosis	SPT/specific IgE	Component resolved diagnosis	Component resolved diagnosis
Lung function	FEV_1_ ≥ 70%	FEV_1_ < 70%	FEV_1_ ≥ 70%	FEV_1_ < 70%	FEV_1_ ≥ 70%	FEV_1_ < 70%	FEV_1_ < 70%	FEV_1_ ≥ 70%
Clinical control of allergic asthma	Controlled	Controlled	Controlled	Not controlled	Partially controlled	Partially controlled	Controlled	Not controlled
Current main treatment of choice for moderate to severe allergic asthma	High dose ICS + LABA^a^	High dose ICS + LABA + biologic therapies	High dose ICS + LABA + biologic therapies	High dose ICS + LABA^a^	High dose ICS + LABA^a^	High dose ICS + LABA + biologic therapies	High dose ICS + LABA^a^	High dose ICS + LABA + biologic therapies

ICS, inhaled corticosteroids; FEV_1_, Forced Expiratory Volume in 1 second; LABA, long-acting beta agonist; SPT, skin prick test.

a Other controlling drugs could be included.

In the context of allergic diseases and asthma, *biologic therapies* refer to monoclonal antibodies directed against specific targets of the type 2 inflammatory response. These include omalizumab (anti-IgE), mepolizumab, reslizumab, and benralizumab (anti-IL-5 or anti-IL-5R), dupilumab (anti-IL-4Rα, blocking IL-4/IL-13 pathways), and tezepelumab (anti-TSLP). Such agents have demonstrated efficacy in reducing exacerbations and improving disease control in subgroups of patients with severe eosinophilic or allergic asthma (13–16). Within the field of allergen immunotherapy, biologic therapies are also considered as potential adjuvant tools to modulate the allergic response, particularly in polysensitized patients or those with severe comorbidities.

### Patient profiles

Using the fractional factorial analysis technique (specifically, the Orthoplan procedure in SPSS), eight eligible patient profiles for AIT were defined from the combination of attributes and their levels. This technique ensured the selected profiles preserved the principle of orthogonality.

### Questionnaire structure

The final questionnaire included four sections: 1) questions regarding participants' experience and the management of moderate to severe allergic asthma in current clinical practice; 2) variables related to opinion regarding the AIT prescription in adult patients with moderate to severe allergic asthma, 3) a single question regarding the importance of characteristics for patient with moderate to severe allergic asthma eligible for AIT; and 4) eight cards containing simulated patient profiles to be classified in order of relevance. Since the order of profile presentation may skew the results, cards presentation was performed randomly for each participant.

The final questionnaire and patient profiles cards can be found in Supplementary Appendix 1.

### Statistical methods and analysis

#### Data collection

Participants completed an online questionnaire specifically designed for this project to capture the variables of interest. Data collection was performed through a unique link for each participant, associated with a user ID that ensured data anonymity and confidentiality.

The key aspects of the adult patient with moderate to severe allergic asthma eligible for AIT were evaluated on a 1–10 scale, being 1 totally disagree, and 10 totally agree. Regarding the patient profile cards, allergists ranked the profiles according to their preferences, from 1 to 8; being 1 the first preference and 8 the last preference.

#### Statistical analysis

A descriptive analysis of quantitative and qualitative variables was carried out. Variables were expressed as frequencies and percentages, measures of central tendency (mean and median), standard deviation (SD), and calculation of 95% confidence intervals (95% CI). To compare contingency tables, a bivariate analysis was performed using the Chi-square test or Fisher's test, when the application of the Chi-square test was not appropriate. For continuous normal variables, *t*-tests for independent samples or analysis of variance were performed depending on the number of groups to be compared, and Wilcoxon Rank-Sum test or Kruskal–Wallis test were used for non-parametric estimations, respectively.

For variables related to opinion, participants used a 9-level scale, being 1 totally disagree and 10: totally agree. Mean results were divided into five categories: 1–2 (strong disagreement), 3–4 (disagreement), 5–6 (neutral), 7–8 (agreement) and 9–10 (strong agreement).

For the analysis of preferences, the CVA approach was used, a multivariate technique that consisted of finding a set of values, called partial utilities or “part-worths”, that associates the levels of the patients' attributes with the preferences. Preference analysis ranged utilities between 0 and 2 according to the subjective value assigned by the participant allergists. Data were analyzed with IBM SPSS, and *p* < 0.05 was considered statistically significant.

## Results

### Participants characteristics

Ninety-one allergists participated in the CVA questionnaire, ranking eight simulated patient profiles eligible for Allergen Immunotherapy (AIT). A full description can be found in Table 2.

**Table 2:** Characteristics of participating allergists.

Characteristics	*N* = 91
Geographical distribution, *n* (%)	
Andalusia	13 (14.3%)
Aragon	3 (3.3%)
Balearic Islands	2 (2.2%)
Basque Country	1 (1.1%)
Canary Islands	5 (5.5%)
Castile - La Mancha	5 (5.5%)
Castile and León	1 (1.1%)
Catalonia	16 (17.6%)
Ceuta	1 (1.1%)
Community of Madrid	11 (12.1%)
Extremadura	2 (2.2%)
Galicia	4 (4.4%)
La Rioja	1 (1.1%)
Melilla	1 (1.1%)
Navarre	1 (1.1%)
Region of Murcia	2 (2.2%)
Valencian Community	7 (7.7%)
Centro Region	3 (3.3%)
Grande Lisboa Region	7 (7.7%)
Norte Region	5 (5.5%)
Workplace, *n* (%)	
Primary	5 (5.5%)
Secondary	20 (22.0%)
Tertiary	66 (72.5%)
Years of experience, *n* (%)	
<5 years	5 (5.5%)
5–10 years	10 (11.0%)
11–15 years	26 (28.6%)
16–30 years	37 (40.7%)
>30 years	13 (14.3%)
Patients with moderate to severe asthma visited in one month, *n* (%)	
<20 patients	11 (12.1%)
20–25 patients	22 (24.2%)
25–30 patients	15 (16.5%)
30–35 patients	5 (5.5%)
>35 patients	38 (41.8%)
Current treatment with AIT, mean (SD)	
Patients with moderate to severe asthma receiving AIT	52.2 (23.1)
Patients with moderate to severe asthma not receiving AIT	47.8 (23.1)

AIT, allergen immunotherapy; SD, standard deviation.

#### Experience

41% had between 16 and 30 years of experience and up to 14.3% reported >30 years of experience.

#### Patient volume

Most participating allergists (88%) reported attending >20 patients per month with an average volume of 35 patients per month.

#### Workplace

The majority of participating allergists (72.5%) worked in tertiary centers. The study included participants across the Iberian Peninsula with the most populated regions, having the highest number of participants Catalonia (17.6%), Andalusia (14.3%) and Madrid (12.1%).

#### AIT prescription

Panelists reported that, on average, 52% of their patients with moderate to severe allergic asthma received AIT, compared to 47.8% who did not.

### Opinion variables

The experts were asked their level of agreement (on a 1–10 scale, where 10 is totally agree) regarding different statements in AIT prescription. The participants agreed on recommending the prescription of AIT if it is associated with allergic rhinitis and presents FEV_1_ ≥ 70%, and if symptoms are controlled with high-dose ICS. In patients treated with biologic therapies and with suboptimal disease control, no conclusive agreement was achieved, although the median results showed a trend towards AIT prescription in these profiles (Table 3).

**Table 3:** Statements related to patients with moderate to severe allergic asthma candidates for AIT (*n* = 91).

Statement	Mean	Median	SD
In adult patients with moderate to severe allergic asthma, the prescription of AIT is recommended if it is associated with allergic rhinitis and the patients presents a FEV_1_ ≥ 70%.	8.5	9	1.8
In adult patients with moderate to severe allergic asthma, the prescription of AIT is recommended if symptoms are controlled with high-dose ICS.	8	8	2.3
In adult patients with moderate to severe allergic asthma, the prescription of AIT is recommended if the symptoms of asthma are controlled with biologic therapies.	6.8	7	2.6
In adult patients with moderate to severe allergic asthma, the prescription of AIT is recommended if there is, at least, a suboptimal control of allergic asthma.	5.7	7	3.4

AIT, allergen immunotherapy; ICS, inhaled corticosteroids; FEV_1_, Forced Expiratory Volume in 1 second; SD, standard deviation. (1: totally disagree - 9: totally agree).

The level of agreement with prescribing AIT when asthma symptoms are controlled with biologic therapies was analyzed based on different subgroups (Supplementary Table S1):

#### Allergists with longer experience vs. allergists with shorter experience

A clear inverse relationship was observed between the participants' years of experience and their agreement to prescribe AIT when the patient's asthma symptoms were controlled with biologic therapies. Experts with longer experience reported lower agreement regarding this combined therapeutic strategy.

#### Allergists working in primary care vs. allergists working in secondary/tertiary settings

Allergists working in primary care reported higher agreement with the recommendation to introduce AIT after achieving asthma control with biologic therapies, compared to their counterparts working in secondary or tertiary centers.

### Classification of attributes according to clinical relevance

When asked about the most relevant criteria individually, the allergists participating in the study prioritized the etiological confirmation of moderate-severe allergic asthma as the criterion with the greatest clinical relevance for prescribing ITA (median position of 1). The next most clinically relevant criteria were clinical control of allergic asthma (median position of 2) and lung function (median position of 3), highlighting the importance given to adequate control of the disease. The criterion to which they assigned the least clinical relevance when evaluating the attributes separately is current main treatment (median position of 4), see results in Supplementary Table S2.

### Conjoint value analysis: expert preferences for AIT eligibility

**Figure 1:**
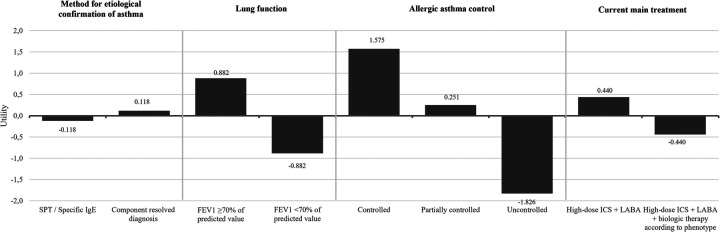
Conjoint value analysis: expert preferences for AIT eligibility in patients with moderate to severe allergic asthma. ICS, inhaled corticosteroids; FEV_1_, Forced Expiratory Volume in 1 second; LABA, long-acting *β*_2_ agonist.

The allergists were asked to rank the four attributes (lung function, clinical control of allergic asthma, current main treatment and method for etiological confirmation) based on their clinical relevance in the context of different patient profiles with moderate to severe allergic asthma, using a patient profile card-sorting exercise. There was consensus among experts that control of allergic asthma was the most important attribute to be considered when choosing AIT (51.6%), followed by lung function (25.0%) (Supplementary Figure S1). According to the results, the levels providing greater utility for identifying patients with moderate to severe allergic asthma candidates to receive AIT were “total control of allergic asthma” (1.575) or partial control (0.251), “lung function FEV_1_ ≥ 70% of predicted value” (0.882), current main treatment based on high-dose ICS + LABA (0.440) and component resolved diagnosis (0.118) (Figure 1). A Pearson's R (0.996) and Kendall's Tau coefficient (1.000) were obtained, indicating a good estimate of the partial utilities (*p* < 0.05) associated with each level of each attribute.

This CVA confirmed that the most important criteria for experts were having good control of the underlying asthmatic condition and preserved lung function (FEV₁ ≥ 70%).

## Discussion

The results of this conjoint analysis, based on the opinions of a group of allergists, showed that clinical control of allergic asthma and lung function are the more relevant attributes to be considered for prescribing AIT. The results of the present study suggest that AIT may be considered a therapeutic option for patients with controlled or partially controlled moderate to severe allergic asthma and preserved lung function (FEV_1_ ≥ 70%). In patients treated with biologic therapies and patients with suboptimal disease control, no conclusive agreement was achieved.

The pathophysiological mechanism of allergic asthma starts with exposure to otherwise harmless antigens, which induces type 2 immune responses. This leads to the infiltration of T helper (Th2) cells to the lung tissue, and it drives an IgE response (17). Since IgE is the key mediator in allergic asthma, it has become an important therapeutic target. On this basis, AIT has been developed as a therapeutic treatment for established IgE-mediated hypersensitivity to common allergen sources. AIT induces a shift from Th2 to Th1 response eventually leading to IgE suppression (5, 7). Therefore, AIT has a true effect on disease cause, with both an immune modifying effect and long-term efficacy on allergic response, slowing the progression of allergic asthma and preventing the worsening of existing respiratory pathology (5, 7). To date, AIT is the only etiological treatment capable of modifying the natural course of the disease, thus representing a real therapeutic alternative for specific allergic asthma patients.

In this framework, the present survey was designed to gather and analyze the opinions of a panel of expert allergists to identify the most relevant real-world attributes for the administration of AIT, independent of the clinical (symptomatic) severity score of allergic asthma.

Current international guidelines for the management of allergic asthma primarily consider the risk of developing an anaphylactic reaction as the key criterion for AIT eligibility. This threshold effectively excludes a large proportion of patients with moderate to severe allergic asthma from AIT. However, literature data indicate that only about 1% of patients—fewer in adults—would experience such a potentially severe reaction upon AIT administration.

The common position emerged from the survey/conjoint analysis presented here is based on the opinions clinical practise of a group of 91 expert Allergists operating in most populated cities of the Iberian Peninsula. In detail, this panel of experts' points out that for the choice of AIT prescription and administration, the ideal patient profile includes the following attributes:

1. Well-controlled or partially controlled moderate to severe allergic asthma;
2. Lung function preserved (FEV1 ≥ 70%);
3. Baseline treatment for asthma (of any type, with or without biologic therapies);
4. Regimen treatment appropriate to asthma control;
5. Confirmation of the allergic aetiology of asthma, regardless the diagnostic method used (STP or CRD).

Furthermore, the sensitization pattern is a critical factor in patient selection and the formulation of AIT. Although the ideal clinical scenario involves monosensitized patients—who generally show a more robust and predictable response, since exposure and immune stimulation are focused on a single clinically relevant allergen (18, 19)—this situation is relatively uncommon in real-world practice. In contrast, polysensitization is highly prevalent and poses additional challenges, as not all allergens detected by skin testing or specific IgE are clinically relevant (20, 21). In such cases, identification of the dominant, causative allergen becomes essential to design an effective AIT regimen and to avoid excessively complex or clinically unsubstantiated treatment schemes (5).

Evidence suggests that AIT can also be effective in polysensitized patients, provided that clinically relevant allergens are selected and prioritized (21). Furthermore, novel strategies—such as the use of standardized extracts targeting multiple allergens or recombinant allergens—have emerged as promising approaches to better address polysensitization (19). In line with this, the EAACI Guidelines emphasize that only clinically relevant allergens, identified through history, skin testing, or specific IgE, should be included in immunotherapy extracts, while indiscriminate mixing of unrelated allergens should be avoided to maintain both efficacy and safety (22). Taken together, these considerations underline the importance of tailoring AIT to the sensitization pattern, thereby optimizing patient selection and maximizing clinical benefit (5).

The emerging perspectives reported here highlight that the confirmation itself holds more value than the technique by which it is achieved (23). Furthermore, no definitive agreement was reached on the prospect of adding AIT to patients with severe allergic asthma treated with biologic therapies. AIT is not recommended in patients with poor disease control and FEV₁ < 70%.

Notably, we found that the duration of clinical practice is a factor influencing the propensity of allergists to prescribing AIT to patients already receiving biologic therapy in moderate to severe allergic asthma. The experts with longer practice years reported lower agreement regarding the AIT prescription following biologic therapies for controlling asthma symptoms. In our opinion, this position shows confidence with the traditional symptomatic therapeutic approaches, possibly driven by clinical habits. Also, risk-benefit perceptions and cautiousness in adopting treatments evolving strategies for disease modification that might have been armored by negative AIT outcomes with poorly standardized/characterized allergen extracts used in the past and an inappropriate route of administration.

In addition, allergists working in primary care reported higher agreement with the recommendation to introduce AIT after achieving asthma control with biologic therapies, compared to their counterparts in secondary or tertiary care settings. This position may reflect higher patient case complexity, access to specialized diagnostic tools, or institutional protocols influencing the sequence of treatments. These differences highlight the need for harmonized clinical guidelines and further real-world evidence to support the optimal integration of AIT in patients with biologic-controlled moderate to severe allergic asthma. Furthermore, professional education efforts could help harmonize clinical practice across different generations of clinicians, ensuring that therapeutic decisions are consistently aligned with both evidence and patient-specific factors.

National and international guidelines on severe asthma specifically recommend IgE testing or SPT for those with severe asthma (8, 24). However, in this study, a low value was assigned to the method used for the etiological confirmation of moderate to severe allergic asthma. This implies that, when a suggestive clinical history is present, the specific diagnostic method used is not considered a key factor in deciding whether AIT is appropriate in patients with severe asthma. In clinical practice, allergists apparently value component-resolved diagnostics more highly than standard IgE testing; however, it was not prioritized in the study's decision-making framework.

The efficacy and safety of AIT in severe allergic asthma has been broadly studied; however, further research on the efficacy and safety of AIT combined with biologic therapies is still needed. Additionally, studies analyzing the impact of AIT beyond symptom control may be useful to determine asthma-related burden, not only from the patient's point of view, but also from a socioeconomical perspective.

Recent real-world evidence from the SAGITAL study has further confirmed that AIT can be both safe and effective in patients with well-controlled severe allergic asthma, showing significant improvements in lung function, quality of life, and a reduction in rescue medication use and emergency visits (25). These results indicate that, although current international guidelines still consider severe asthma a contraindication for AIT, in clinical practice immunotherapy is already being prescribed to carefully selected patients outside of guideline recommendations. In this context, our conjoint analysis adds value by systematically identifying the patient profiles that allergists themselves consider as candidates for AIT. The convergence of findings highlights the pressing need to move beyond individual clinical decisions and develop clear, evidence-based guidelines to ensure that AIT can be administered more safely and consistently in this complex population.

### Limitations

The present study had several methodological limitations. The participants were invited to participate and responded voluntarily. Therefore, the collected results may not be fully representative of the total population of experts (26). Furthermore, the conclusions must be validated in clinical practice. Finally, the allergists participating in the study were representative of the clinical practice across Spain and Portugal reflecting the vision for management including a populous European country, whether the conclusions of this study can be extrapolated to other countries or cultural settings is unclear.

## Conclusion

The results of this study may improve asthma management by underscoring the ideal patient profile with moderate to severe allergic asthma as candidate for AIT. The findings emphasize that well-controlled asthma and preserved lung function are key prerequisites for initiating AIT, reflecting a cautious but open stance toward its use in this population. Nevertheless, further studies exploring this possibility are still needed. Importantly, while the type of main asthma treatment and the method of etiological confirmation are seen as secondary, the need for asthma control and confirmed allergic sensitization remains central. Altogether, these perspectives reflect a shift toward a more individualized, risk-aware integration of AIT in the therapeutic strategy for severe allergic asthma, an approach that could ultimately expand access to AIT and enhance patient outcomes.

## Data Availability

The raw data supporting the conclusions of this article will be made available by the authors, without undue reservation.
