# Supplementary material for: Allergen immunotherapy for the control of moderate to severe allergic asthma: an evidence-based conjoint analysis to define candidate patient profiles in Spain and Portugal
Source: Front Allergy. 2025 Oct 30;6:1676399. doi: 10.3389/falgy.2025.1676399 (PMC12611875; doi:10.3389/falgy.2025.1676399)
Supplement: Supplementary file 1 [file Datasheet1.docx]

Supplementary Material

**Appendix 1.** Questionnaire and patient profiles cards.

**Supplementary Figure 1.** Relative importance of attributes.

**Supplementary Figure 2.** Utility of attributes according to the percentage of patients visited in one month.

**Supplementary Figure 3.** Preferences according to the percentage of patients receiving AIT.


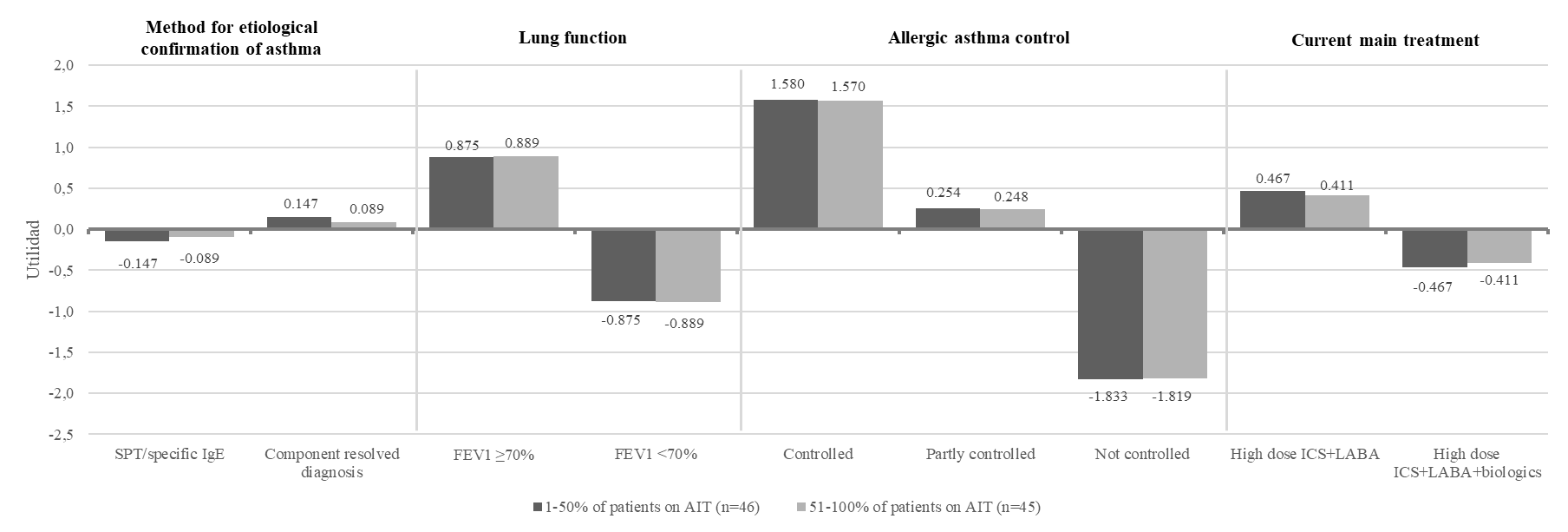


ICS: inhaled corticosteroids; FEV1: Forced Expiratory Volume in 1 second; LABA: long-acting β2 agonist; SPT: skin prick test.

**Supplementary Figure 4.** Utility of attributes according to the percentage of patients with allergic asthma visited in one month.

9.80%

9.99%

14.27%

12.68%

26.75%

23.05%

49.18%

54.28%

**Supplementary Figure 5.** Preferences according to the percentage of patients with allergic asthma visited in one month.


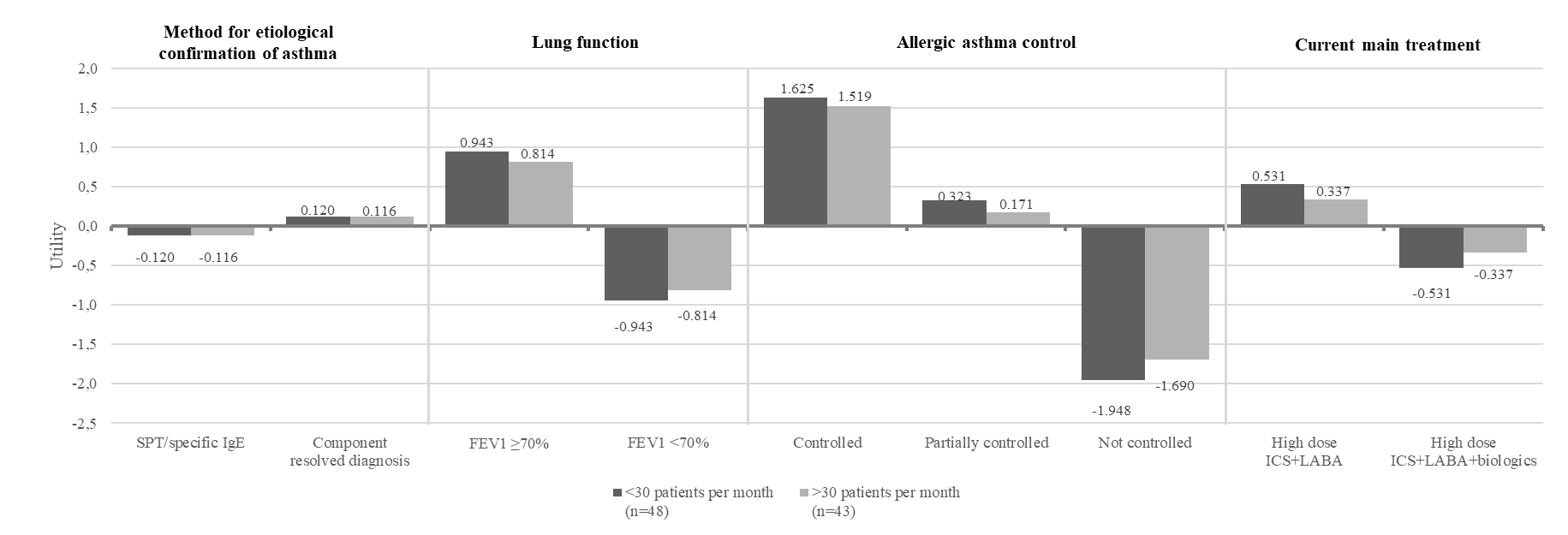


ICS: inhaled corticosteroids; FEV_1_: Forced Expiratory Volume in 1 second; LABA: long-acting β_2_ agonist; SPT: skin prick test.

**Supplementary Table 1.** Statements related to patients with moderate-severe allergic asthma candidates for AIT according to years of experience and workplace of the panelists (n=91)

| In adult patients with moderate-severe allergic asthma, the prescription of AIT is recommended if… | **Workplace** | | | **Years of experience** | | | | |
| --- | --- | --- | --- | --- | --- | --- | --- | --- |
|  | Primary | Secondary | Tertiary | <5 years | 5-10 years | 11-15 years | 16-30 years | >30 years |
| … **there is, at least, a suboptimal control of allergic asthma**, (n) | 5 | 20 | 66 | 5 | 10 | 26 | 37 | 13 |
| Mean (SD) | 6.0 (4.2) | 5.5 (3.1) | 5.7 (3.4) | 6.2 (3.6) | 5.7 (3.8) | 5.2 (3.3) | 5.5 (3.5) | 6.8 (2.6) |
| **…the symptoms of asthma are controlled with biologics**, (n) | 5 | 20 | 66 | 5 | 10 | 26 | 37 | 13 |
| Mean (SD) | 8.0 (1.6) | 6.7 (2.8) | 6.7 (2.6) | 7.8 (1.5) | 7.9 (2.1) | 7.1 (2.7) | 6.6 (2.6) | 5.2 (2.4) |
| **…symptoms are controlled with high-dose ICS**, (n) | 5 | 20 | 66 | 5 | 10 | 26 | 37 | 13 |
| Mean (SD) | 7.8 (3.3) | 8.1 (1.5) | 8.0 (2.5) | 9.0 (1.4) | 8.6 (1.6) | 8.3 (1.9) | 7.6 (2.6) | 7.5 (2.8) |
| **…it is associated with allergic rhinitis and presents a FEV_1_≥70%**, (n) | 5 | 20 | 66 | 5 | 10 | 26 | 37 | 13 |
| Mean (SD) | 8.2 (3.0) | 8.5 (1.7) | 8.6 (1.8) | 9.6 (0.9) | 9.2 (1.1) | 8.4 (1.9) | 8.4 (2.0) | 8.5 (2.0) |

AIT: allergen immunotherapy; ICS: inhaled corticosteroids; FEV_1_: Forced Expiratory Volume in 1 second; SD: standard deviation.

**Supplementary Table 2**. Descriptive analysis of the results obtained in the ranking of selected attributes according to their clinical relevance.

| **Attribute** | **n** | **Mean** | **Median position** | **SD** |
| --- | --- | --- | --- | --- |
| Method for ethiological confirmation of asthma | 91 | 1,7 | 1 | 1,1 |
| Allergic asthma control | 91 | 2,0 | 2 | 0,7 |
| Lung function | 91 | 2,9 | 3 | 0,8 |
| Current main treatment | 91 | 3,5 | 4 | 0,8 |
